# A four-state adaptive Hopf oscillator

**DOI:** 10.1371/journal.pone.0249131

**Published:** 2021-03-25

**Authors:** XiaoFu Li, Md Raf E Ul Shougat, Scott Kennedy, Casey Fendley, Robert N. Dean, Aubrey N. Beal, Edmon Perkins

**Affiliations:** 1 Department of Mechanical & Aerospace Engineering, North Carolina State University, Raleigh, NC, United States of America; 2 Department of Electrical & Computer Engineering, Auburn University, Auburn, AL, United States of America; 3 Department of Electrical & Computer Engineering, University of Alabama in Huntsville, Huntsville, AL, United States of America; Lanzhou University of Technology, CHINA

## Abstract

Adaptive oscillators (AOs) are nonlinear oscillators with plastic states that encode information. Here, an analog implementation of a four-state adaptive oscillator, including design, fabrication, and verification through hardware measurement, is presented. The result is an oscillator that can learn the frequency and amplitude of an external stimulus over a large range. Notably, the adaptive oscillator learns parameters of external stimuli through its ability to completely synchronize without using any pre- or post-processing methods. Previously, Hopf oscillators have been built as two-state (a regular Hopf oscillator) and three-state (a Hopf oscillator with adaptive frequency) systems via VLSI and FPGA designs. Building on these important implementations, a continuous-time, analog circuit implementation of a Hopf oscillator with adaptive frequency and amplitude is achieved. The hardware measurements and SPICE simulation show good agreement. To demonstrate some of its functionality, the circuit’s response to several complex waveforms, including the response of a square wave, a sawtooth wave, strain gauge data of an impact of a nonlinear beam, and audio data of a noisy microphone recording, are reported. By learning *both* the frequency and amplitude, this circuit could be used to enhance applications of AOs for robotic gait, clock oscillators, analog frequency analyzers, and energy harvesting.

## 1 Introduction

Adaptive oscillators are similar to phase-locked loops, except they have an additional, direct injection of the external forcing on the oscillator itself. Previously, only two circuit implementations of adaptive oscillators have been reported, both of which were Hopf Adaptive Frequency Oscillators (HAFOs)—a VLSI implementation (noting that the VLSI implementation did not report any experimental results) [1] and an FPGA implementation [2]. The two primary goals for this manuscript are 1) to provide a HAFO architecture with an additional plastic state that builds on these important, previous contributions and 2) to report new hardware results for a physical implementation. As few published designs currently exist and verified hardware measurements are scarce in the literature, this second point is especially important.

Early studies of adaptive phase oscillators were used as neural models to reproduce sinusoidal inputs [3]. More generally, adaptive oscillators (AOs) can be created by adding additional, plastic states to a nonlinear oscillator [4]. The derivation of adaptive frequency oscillators (AFOs) was originally based on a dynamic Hebbian learning rule implemented in hardware by modifying the Hopf system [5]. The correlation-based Hebbian rule was inspired from the firing rate of neurons, such that the information transmission between different neurons will be increased due to electrical activity, which can synchronize neural networks [6]. Generally, learning characteristics of AOs can be preferable when compared with their counterparts, such as the relaxation oscillator and the phase oscillator [7, 8], which are limited to simple inputs and have smaller basins of attraction.

Several practical applications of AOs have been explored. *Central pattern generators* (CPGs) have been used to control the motion of individual joints of walking robots [9], and adaptive frequency oscillators can work together to create an arbitrary CPG [10–12]. Various gait patterns for locomotion can be achieved through assigning specific frequency and phase angles to individual oscillators in an array. Applications for locomotion control using Field Programmable Gate Arrays (FPGAs) with the aid of CPGs and coupled nonlinear oscillators have also been proposed [13–15]. Specifically, the HAFO has been used for robotic locomotion control by employing HAFOs as CPGs to tune walking patterns in a cooperative way [16–18]. Body dynamics of these robots often vary over time due to obstacles and environmental fluctuations. These oscillators can be used as adaptive controllers that find and match intrinsic frequencies leading to enhanced energy efficiency of the resulting gait [19, 20]. Additionally, AFOs have been proposed to detect gait phase and frequency in robots for assisting people with walking difficulties due to muscle weakness [21] or reducing the metabolic cost of walking [22]. State observer AFOs have been used to create cross adaptation between users and assistance robots resulting in reduced effort supporting arm muscles’ activities [23]. An array of AOs has also been proposed as a biologically-inspired analog signal analyzer [24]. This form of analog signal analyzer subtracts the dynamic response of AOs from an input signal, which causes the array’s frequency states to converge to the frequencies present in the input signal [25].

Considering these mentioned uses, an AO that is capable of learning additional states could be useful to many applications. In this paper, a four-state adaptive oscillator analog circuit that links theory discussed by [5] is presented. The resulting system provides the dynamics of 1) A two-state regular Hopf oscillator, 2) A three-state AO that can learn the *frequency* of an input sinusoid, and 3) A four-state AO that can learn the *frequency* and *amplitude* of an input sinusoid and extends the work of [1, 2]. These schemes are similar to AOs that have previously improved phase-locked loop designs through Lyapunov treatments [26]. In this letter, we show a topology that extends the number of adaptive states previously implemented and show direct injection of a forcing signal. This AO is similar to the phase-locked loop Lyapunov design [26]; in the Lyapunov design, the forcing frequency needed to be directly inputted into the system. In the current AO, the forcing signal is injected directly into the *x*, *ω*, and *α* states without knowledge of the frequency. This is an important distinction, since the forcing signal, *a* sin(Ω*t* + *ϕ*_0_), is available, while the forcing frequency, Ω, might not be precisely known at a specific time.

In the following sections, a standard Hopf oscillator is described. Then, plastic states are progressively added to the Hopf equations by modifying the system dynamics as reported in [5, 10]. This treatment results in 1) the addition of a fourth, plastic state and 2) three systems that inform circuit designs based on state variable networks.

## 2 Two-state system

The Hopf oscillator is a nonlinear oscillator described by the following ordinary differential equations (ODEs) [27]:

$$\begin{array}{c} \begin{array}{c} \dot{x}=\left(\mu -\left(x^{2}+y^{2}\right)\right)x-\omega_{0}y+k\left(a\;\text{sin}\left(\Omega t+\phi_{0}\right)\right)\\ \dot{y}=\left(\mu -\left(x^{2}+y^{2}\right)\right)y+\omega_{0}x \end{array} \end{array}$$ (1)

Here, *ω*_0_ is a resonance constant, *μ* is a constant that controls the limit cycle radius, and *k* is a coupling constant. The input signal is *a* sin(Ω*t* + *ϕ*_0_), where *a* is the amplitude of the sinusoid, Ω is the external forcing frequency, and *ϕ*_0_ is the phase of the input sinusoid. This nonlinear oscillator is used as the building block for the subsequent adaptive oscillator systems. Since this system is not adaptive, the frequency-amplitude relationship has a single peak, as presented in Fig 5.

## 3 Three-state system

**Fig 1:**
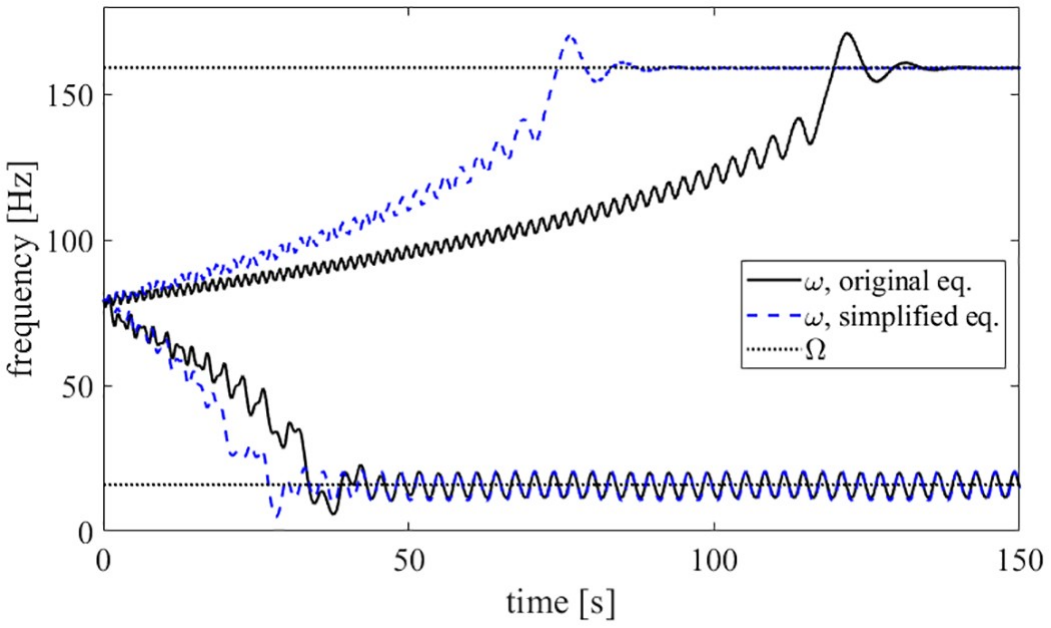
Comparison of original and modified AFOs. For two forcing frequencies, a comparison of the original AFO as proposed by [5] and the simplified system, as given in Eq 2, is shown.

The Hopf Adaptive Frequency Oscillator (HAFO) may learn the frequency of an external stimuli; this implies that the HAFO is a three-state nonlinear oscillator with *dynamical plasticity*. This synchronization process is appealing, as it does not need any pre- or post-processing. Here, *y* is used to replace the term $\frac{y}{\sqrt{x^{2}+y^{2}}}$ that was proposed in [5]. If the constants in Eq 1 are chosen to create a stable limit cycle, this limit cycle will have a constant radius after the transient response decays. This modification simplifies the circuit implementation in a similar manner shown in [1]. Fig 1 compares the originally derived AFO in [5] with the simplifications imposed on Eq 2. After their transient response decays, the outputs of the two systems are qualitatively similar. With this modification, the complexity of the circuit implementation is highly reduced and the learning time of the *ω* state is decreased. The frequency information stored in the third state, *ω*, is achieved by the system’s intrinsic dynamics. The first two ODEs are the classical form of the Hopf oscillator, and the third ODE allows for frequency adaptation. Input forcing causes the resulting HAFO output to increase or decrease in frequency based on a frequency difference referenced to the input signal. The three-state system’s ODEs are given as follows:

$$\begin{array}{c} \begin{array}{c} \dot{x}=\left(\mu -\left(x^{2}+y^{2}\right)\right)x-\omega y+k\left(a\;\text{sin}\left(\Omega t+\phi_{0}\right)\right)\\ \dot{y}=\left(\mu -\left(x^{2}+y^{2}\right)\right)y+\omega x\\ \dot{\omega }=-k\left(a\;\text{sin}\left(\Omega t+\phi_{0}\right)\right)y \end{array} \end{array}$$ (2)

It should be noted that the resonance constant, *ω*_0_, in Eq 1 has been replaced by the state, *ω*. By allowing the resonance frequency to be a state variable instead of a constant, the Hopf oscillator may now learn the external forcing frequency. It should also be noted that the external input signal is injected into both the *x* and the *ω* states.

## 4 Four-state system

By adding another state and modifying the equation for $\dot{x}$ to include *xα* in Eq 2, a four-state oscillator [10] can be created that learns the amplitude of the external signal in addition to frequency. It should be noted that the magnitude of *α* at steady state will deviate from the input amplitude when *μ* ≠ 1. Detailed information concerning the derivation of these equations was reported in [5, 28]; however, few circuit designs have been reported and little hardware verification exists in the literature. The four-state system’s ODEs are given as follows:

$$\begin{array}{c} \begin{array}{c} \dot{x}=\left(\mu -\left(x^{2}+y^{2}\right)\right)x-\omega y+k\left(a\;\text{sin}\left(\Omega t+\phi_{0}\right)-x\alpha \right)\\ \dot{y}=\left(\mu -\left(x^{2}+y^{2}\right)\right)y+\omega x\\ \dot{\omega }=-k\left(a\;\text{sin}\left(\Omega t+\phi_{0}\right)-x\alpha \right)y\\ \dot{\alpha }=\eta \left(a\;\text{sin}\left(\Omega t+\phi_{0}\right)-x\alpha \right)x \end{array} \end{array}$$ (3)

Here, *η* is a coupling constant. It should also be noted that the external input signal is injected into the *x*, *ω*, and *α* states. In the four-state system, Hebbian learning (i.e., subtracting *xα* from the external input signal) is used to enforce that *ω* and *α* converge to the external input signal.

## 5 Local analysis

To gain a better understanding of adaptive oscillators, a local analysis is performed on the Hopf adaptive frequency oscillator (Eq 2). For comparison, this local analysis will first be used on the classical Hopf oscillator (the two-state system represented by Eq 1). Removing the forcing term from this equation, the Jacobian may be written as:

$$J_{1}=[\begin{array}{cc} -3x^{2}-y^{2}+\mu & -2xy-\omega_{0}\\ -2xy+\omega_{0} & -x^{2}-3y^{2}+\mu \end{array}]$$ (4)

For the fixed point $\left(x,y\right)=\vec{0}$, Mathematica was used to find the eigenvalues of this system. The eigenvalues of *J*_1_ are the conjugate pair, *μ*±*ω*_0_ *i*. This is because the unforced Hopf oscillator given by Eq 1 with *μ* > 0 possesses a limit cycle with frequency *ω*_0_ [27].

To perform this analysis for the three-state system, the forcing term, sin(Ω*t* + *ϕ*_0_), is replaced by an additional oscillator in order for the system of equations to be autonomous, as in [29]:

$$\begin{array}{c} \begin{array}{c} \dot{u}=u+\Omega v-u\left(u^{2}+v^{2}\right)\\ \dot{v}=v-\Omega u-v\left(u^{2}+v^{2}\right) \end{array} \end{array}$$ (5)

The system represented by Eq 5 has a supercritical Andronov-Hopf bifurcation [30]. Replacing the forcing term in Eq 2, the following system of equations obtained:

$$\begin{array}{c} \begin{array}{c} \dot{x}=\left(\mu -\left(x^{2}+y^{2}\right)\right)x-\omega y+k\left(au\right)\\ \dot{y}=\left(\mu -\left(x^{2}+y^{2}\right)\right)y+\omega x\\ \dot{\omega }=-k\left(au\right)y\\ \dot{u}=u+\Omega v-u\left(u^{2}+v^{2}\right)\\ \dot{v}=v-\Omega u-v\left(u^{2}+v^{2}\right) \end{array} \end{array}$$ (6)

These equations are autonomous, and their Jacobian may be written as:

$$J_{2}=[\begin{array}{ccccc} j_{1} & j_{2} & j_{3} & j_{4} & 0\\ j_{5} & j_{6} & j_{7} & 0 & 0\\ 0 & j_{8} & 0 & j_{9} & 0\\ 0 & 0 & 0 & j_{10} & j_{11}\\ 0 & 0 & 0 & j_{12} & j_{13} \end{array}]$$

where *j*_1_ = −3*x*^2^ − *y*^2^ + *μ*, *j*_2_ = −2*xy* − *ω*, *j*_3_ = −*y*, *j*_4_ = *ak*, *j*_5_ = −2*xy* + *ω*, *j*_6_ = −*x*^2^ − 3*y*^2^ + *μ*, *j*_7_ = *x*, *j*_8_ = −*aku*, *j*_9_ = −*aky*, *j*_10_ = 1 − 3*u*^2^ − *v*^2^, *j*_11_ = −2*uv* + Ω, *j*_12_ = −2*uv* − Ω, *j*_13_ = 1 − *u*^2^ − 3*v*^2^. For the fixed point $\left(x,y,u,v\right)=\vec{0}$, the eigenvalues for the *J*_2_ are 1 ± Ω*i*, *μ* ± *ωi*, and 0. The conjugate pair 1 ± Ω*i* corresponds to the forcing oscillator, with forcing frequency Ω. The conjugate pair *μ* ± *ωi* corresponds to the Hopf adaptive frequency oscillator; thus, the Hopf adaptive frequency oscillator oscillates with frequency equal to the third state, *ω*. The eigenvalue of 0 corresponds to the *ω* state. It is not stable or unstable, which allows this state to plastically deform to the forcing frequency.

## 6 Experimental results

### 6.1 Circuit design

**Fig 2:**
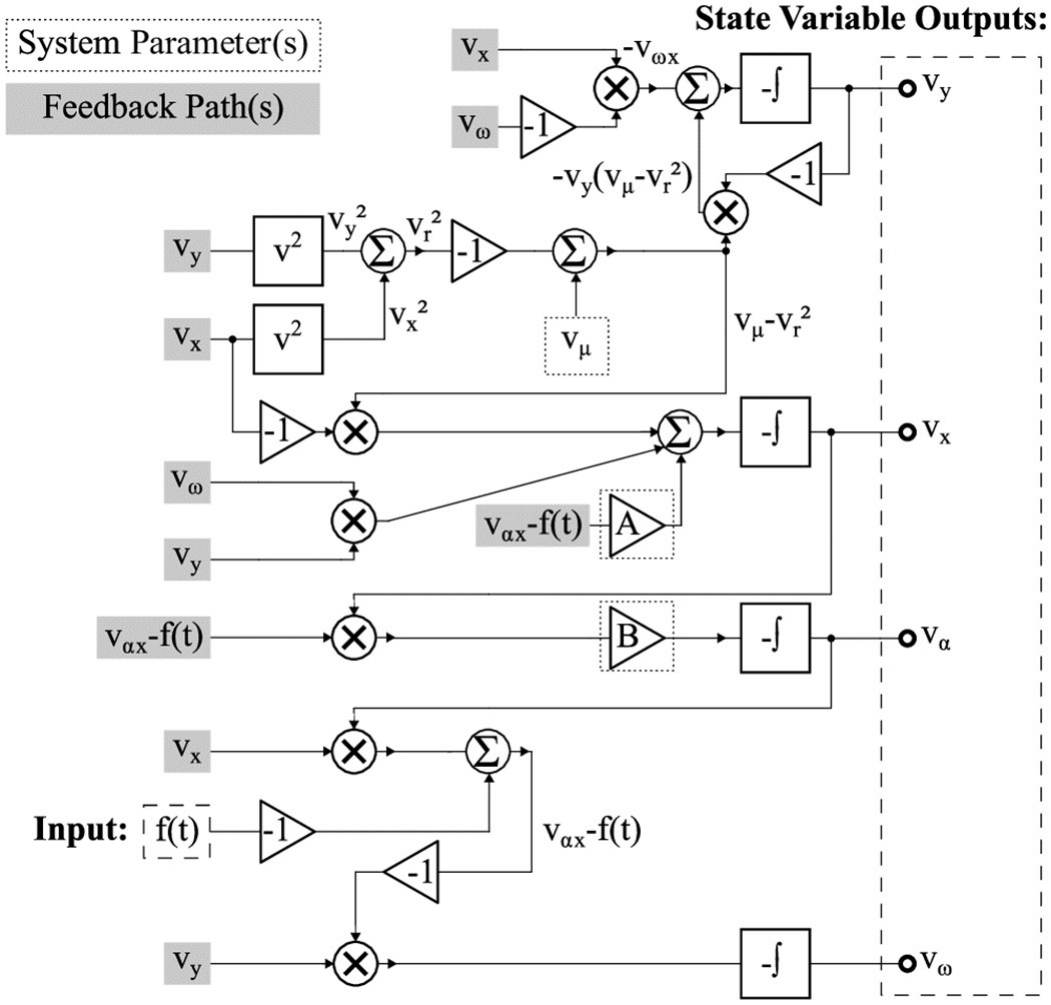
Simplified circuit schematic. A simplified schematic for the four-state adaptive system, with states *V*_*x*_, *V*_*y*_, *V*_*ω*_, and *V*_*α*_.

**Fig 3:**
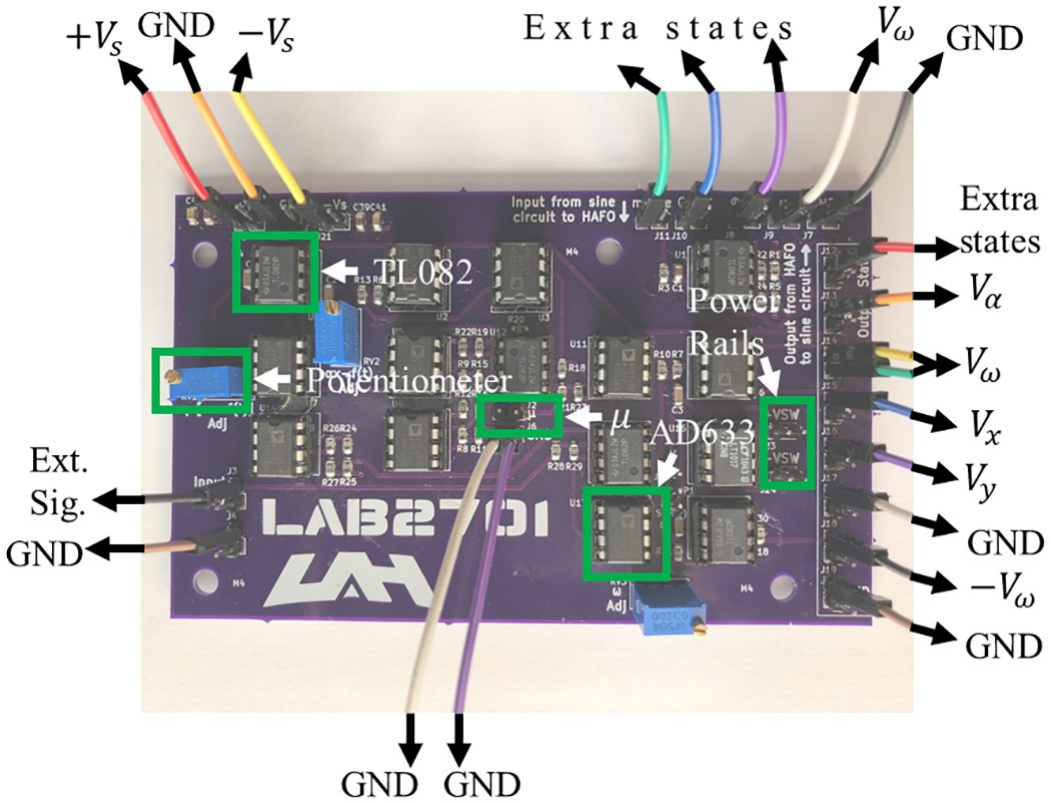
Printed circuit board. PBC used in experiments. Here, “Ext. Sig.” is given by *V*_*P*_(*t*).

A circuit implementation of the system described by Eq 3 was designed, fabricated, and tested. A complete, detailed schematic is shown in S1 Fig. For clarification, a simplified schematic is shown in Fig 2. Three potentiometers, annotated as RV1, RV2 and RV3, are set to 10kΩ, 12.5kΩ, 200kΩ, respectively. Additionally, the tunable resistance ranges of RV1, RV2 and RV3 are from 0Ω to 500kΩ. All unlabeled resistors are 1kΩ. The capacitors, labeled C1, C2, C3, and C4, have a capacitance of 0.1 *μ*F. The experimental PCB is shown in Fig 3.

This implementation was realized using TL082 opamps and AD633 multipliers in standard configurations with 0805 SMD passive components with 1% error tolerance for resistors and 2% error tolerance for capacitors. Three potentiometers were included to tune the coupling strengths associated with the first, third, and fourth states.

The dynamical system in Eq 3 was then mapped to Eq 7. This was done in a manner similar to that discussed in [31], where Kirchhoff’s laws may be used to create a standard, voltage-mode opamp integrator configuration paired with voltage multipliers. This also resembles the analog circuit implementations shown in [32]. where *V*_*P*_(*t*) = [*a* sin(Ω*t* + *ϕ*_0_)) − *xα*] is an external stimulus.

$$\begin{array}{c} \begin{array}{cc} {\dot{V}}_{x}= & -\frac{1}{R1C1}\left(V_{\mu }-\left(V_{x}^{2}+V_{y}^{2}\right)\right)V_{x}+\frac{1}{R2C1}V_{\omega }V_{y}-\frac{1}{RV2C1}V_{P}\left(t\right)\\ {\dot{V}}_{y}= & -\frac{1}{R3C2}\left(V_{\mu }-\left(V_{x}^{2}+V_{y}^{2}\right)\right)V_{y}-\frac{1}{R4C2}V_{\omega }V_{x}\\ {\dot{V}}_{\omega }= & \frac{1}{RV3C3}V_{P}\left(t\right)V_{y}\\ {\dot{V}}_{\alpha }= & -\frac{1}{RV1C4}V_{\eta }V_{P}\left(t\right)V_{x} \end{array} \end{array}$$ (7)

Here, the states *V*_*x*_, *V*_*y*_, *V*_*ω*_, and *V*_*α*_ correspond to the voltage outputs of opamp integrators. This methodology is similar to the realization of integrator-based state variable networks.

**Fig 4:**
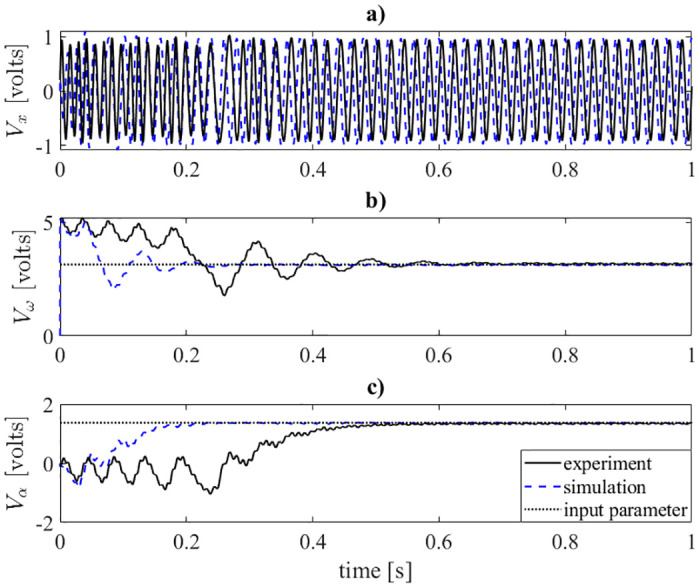
Time history of the four-state oscillator. Experimental data is plotted as a black solid line, SPICE simulation data is plotted as a blue dashed line, and the sinusoid’s Ω and *a* are plotted as dotted lines.

**Fig 5:**
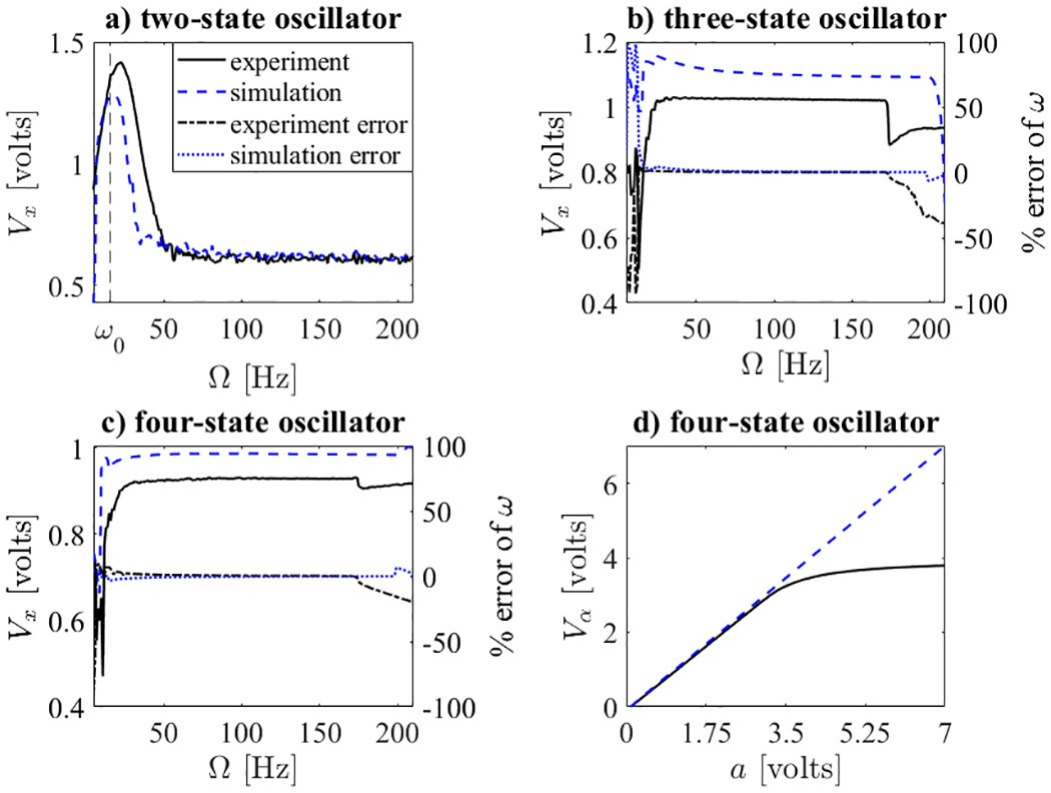
Range of operation. Comparison of results of PCB experiment and SPICE simulations, with *k* = 0.5, *a* = 1.5 (except (d)), *ω*_0_ ≈ 15.9 Hz, and *μ* = 1 for both the simulations and the experiments. It should be noted that the frequency-amplitude relationship is reported for the *x*-state, while the percent error is reported for the *ω*-state. Noting that for the four-state system (bottom-right), the oscillator learns the correct amplitude from approximately 0 to 3; after 3, nonlinear effects in the PCB cause the simulation and experimental results to diverge.

By connecting the output pin headers of the *α* state to the ground, the four-state system can be transformed into the three-state system. This effectively sets the *αx* term in the $\dot{x}$, $\dot{\omega }$, and $\dot{\alpha }$ equations to zero. By connecting the output pin headers of both the *α* state to the ground and the *ω* state to a constant, the four-state system can be transformed into a regular two-state Hopf oscillator. Therefore, the PCB can switch its functionality between a two-state, three-state, and four-state system. The PCB, whose dynamics is represented by Eq 7 was fabricated, and the experimental results from this PCB are depicted in Figs 4 and 5.

### 6.2 Range of operation

The results shown in Fig 4 are the time response of the *x*, *ω*, and *α* states. The results display qualitatively similar characteristics.

The simulation results shown in Fig 5 are a comparison of SPICE simulations and the experimental results of the PCB. It should be noted that no filters were used on any of the results collected from the experiments. In Fig 5, the black solid lines represent experimental data, while the blue dashed lines represent simulation results. The black dash-dotted and blue dotted lines show the percent error of the learned input of the experiment and simulation, respectively.

All the external stimuli for the experimental results were generated with MATLAB and input to the PCB with a National Instruments (NI) cDAQ-9174. In Fig 5(a), the frequency-amplitude response of the two-state Hopf oscillator is shown. A frequency sweep was performed from 5 Hz to 210 Hz. The frequency-amplitude response from the SPICE simulations show qualitatively similar results with slightly different resonance frequency and corresponding magnitude. There is only one resonance peak near *ω*_0_ (which is marked as a vertical dashed line) in the tested frequency range, since it is not an AO. In Fig 5(b), the frequency-amplitude response of the three-state system is shown. Instead of a single resonance, the three-state system maintains a large value over a wide range of frequencies. The percent error is calculated as $\frac{\Omega -<\omega >}{\Omega }\times 100$, where <*ω*> is the mean of the *ω* state at steady state. For the experiment, the error is less than 2% when the forcing frequency is between 19 Hz and 173 Hz. For the simulations, the percent error is less than 3% when the forcing frequency is between 20 Hz and 198 Hz. The frequency-amplitude relationship of the three-state oscillator matches closely with the percent error relationship (i.e., when the amplitude is high, the percent error is small).

In Fig 5(c), the frequency-amplitude response of the four-state system is shown. For the experiment, the percent error is less than 2.5% if the forcing frequency is between 25 Hz and 174 Hz. For the simulations, the percent error displays qualitatively similar behavior. In Fig 5(d), the resulting *α* when sweeping the input amplitude from 0 to 7 for both the simulations and the experiment are shown. The experimental results show that the linear relationships between *a* and *α* is maintained until *a* > 3. A linear curve-fit was performed in MATLAB (for *a* < 3), and the slope of the line was 0.986 for the experiment. For the simulation, the linear relationship was maintained even outside this region, and the slope of the curve-fit was 1.018. Both the experiments and simulations have less than 2% error. It should be noted that the scalar parameter, *μ*, controls the amplitude of *x* at steady state, and a slight deviation of *μ* generated by the power supply from its expected value will cause an error between the experiment and the desired result. Additionally, the nonlinearity of the electrical components used in the simulations are not perfectly modeled, so the resulting differences inevitably occur in Fig 5.

### 6.3 Verification of other waveforms

**Fig 6:**
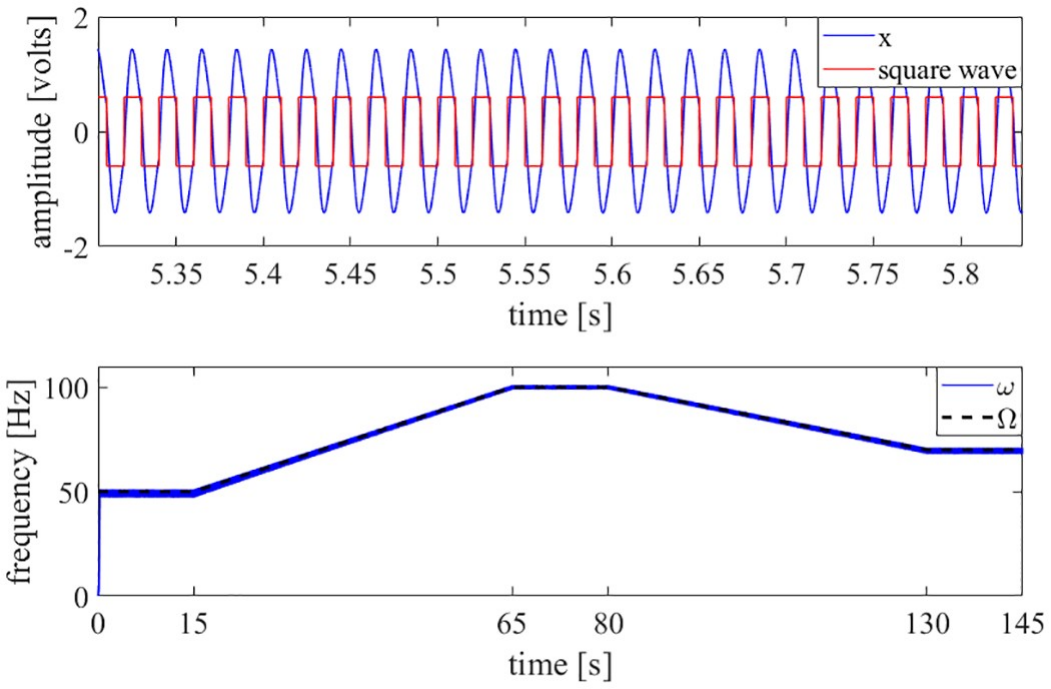
Square wave results. A generated square wave with a varying fundamental frequency was used as the input to the adaptive oscillator circuit. The circuit was able to learn the fundamental frequency of the square wave. Top: The time history of the *x* state and the generated square wave are shown. Bottom: The circuit learns the fundamental frequency of the square wave, even though the frequency was varying over time.

**Fig 7:**
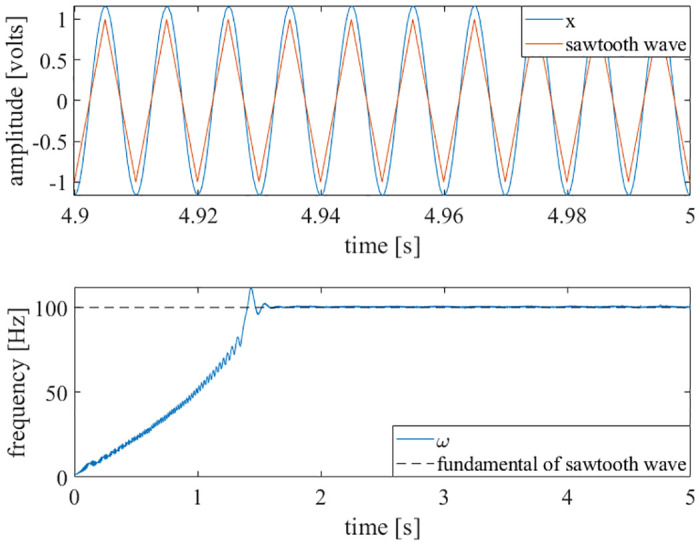
Sawtooth wave results. A generated sawtooth wave was used as the input to the adaptive oscillator circuit. The circuit was able to learn the fundamental frequency of the sawtooth wave. Top: The time history of the *x* state and the generated sawtooth wave are shown. Bottom: The circuit learns the fundamental frequency of the sawtooth wave.

After the initial testing to determine the range of operation of the experimental four-state adaptive oscillator system, the circuit was then tested using several complex waveforms. A signal generator was used to create a square wave and a sawtooth wave; the square wave was constructed so that the fundamental frequency changes over time. These signals are more complex than a simple sinusoidal wave, as they are composed of an infinite series of sinusoids. The results are shown in Figs 6 and 7.

**Fig 8:**
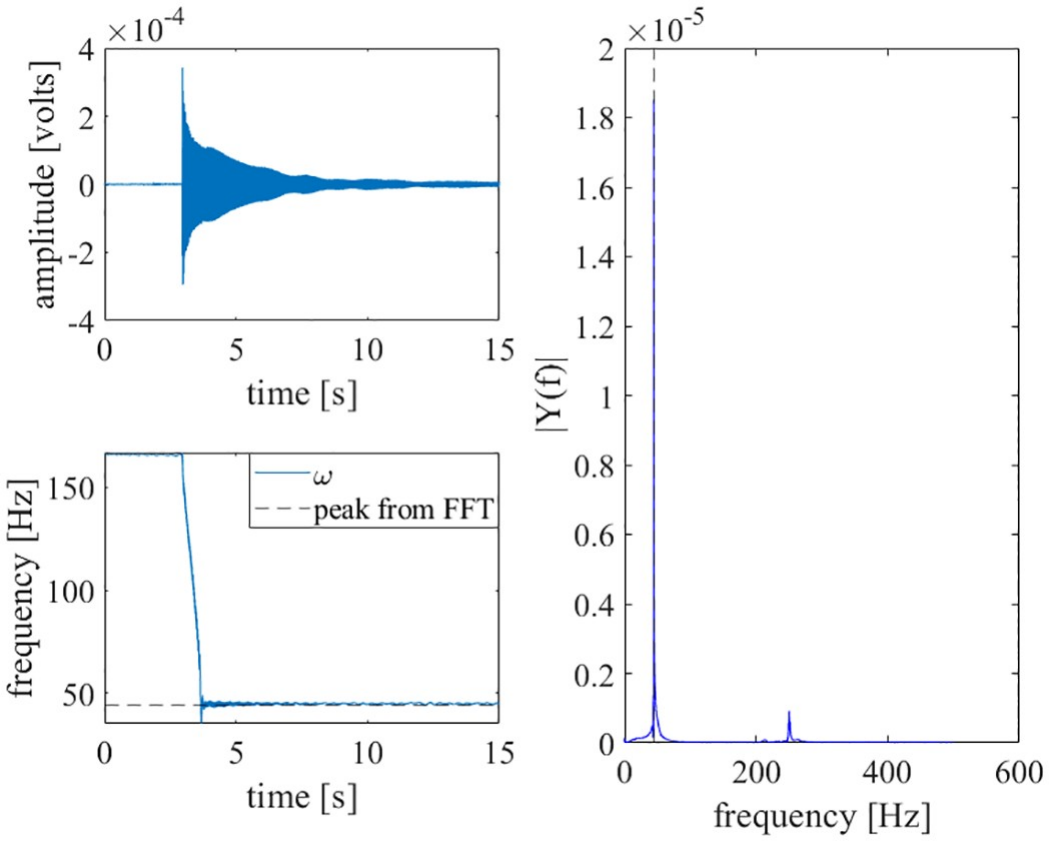
Impact test results. Strain gauge data [33] was directly input to the adaptive oscillator circuit. The circuit learned the natural frequency of the nonlinear beam. Top-left: The time history of the strain gauge’s voltage is shown. The impact of the beam happens at approximately 3 seconds. Bottom-left: The circuit learns the first natural frequency of the beam. Right: An FFT of the beam is shown, with the first natural frequency marked by a dashed, vertical line.

Further, the voltage data from a strain gauge that was mounted to a nonlinear beam was used as an input to the adaptive oscillator circuit. By impacting the beam with a hammer, a vibratory system identification was performed to find the first natural frequency of the beam [33]. The results are shown in Fig 8.

**Fig 9:**
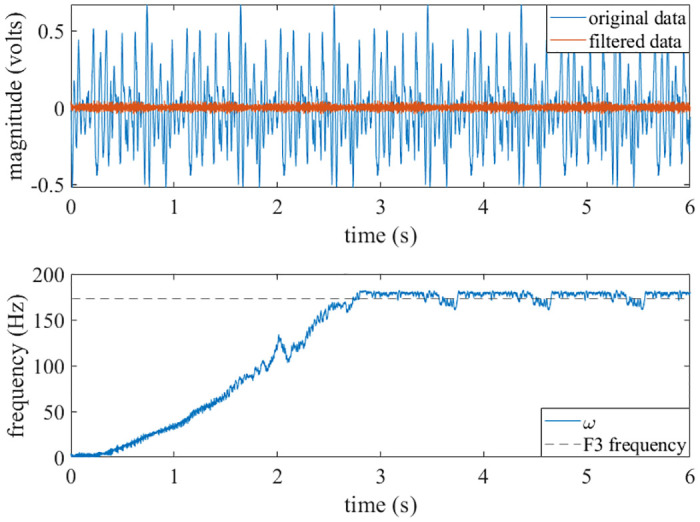
Asphaltophone results. A filtered audio recording of an asphaltophone was input to the adaptive oscillator circuit. Top: The original signal and the filtered signal are plotted. Bottom: The circuit learns the F3 note from the asphaltophone. The data is still quite noisy, so the *ω* state has fluctuations about the F3 frequency.

A vehicle’s response to an asphaltophone was recorded with a microphone [34]. This recording had high amplitude low-frequency noise; the amplitudes of this noise were larger than the signal. In this recording, the asphaltophone was designed to reproduce a note of F3 (174.6141 Hz) when the vehicle is traveling 35 miles per hour. As the note is rather short, it was concatenated to create a longer signal. The low-frequency noise was filtered from the recording, and the filtered recording was input to the circuit. It should be noted that this example is the only time that filtering was used in this paper. The results are shown in Fig 9.

**Fig 10:**
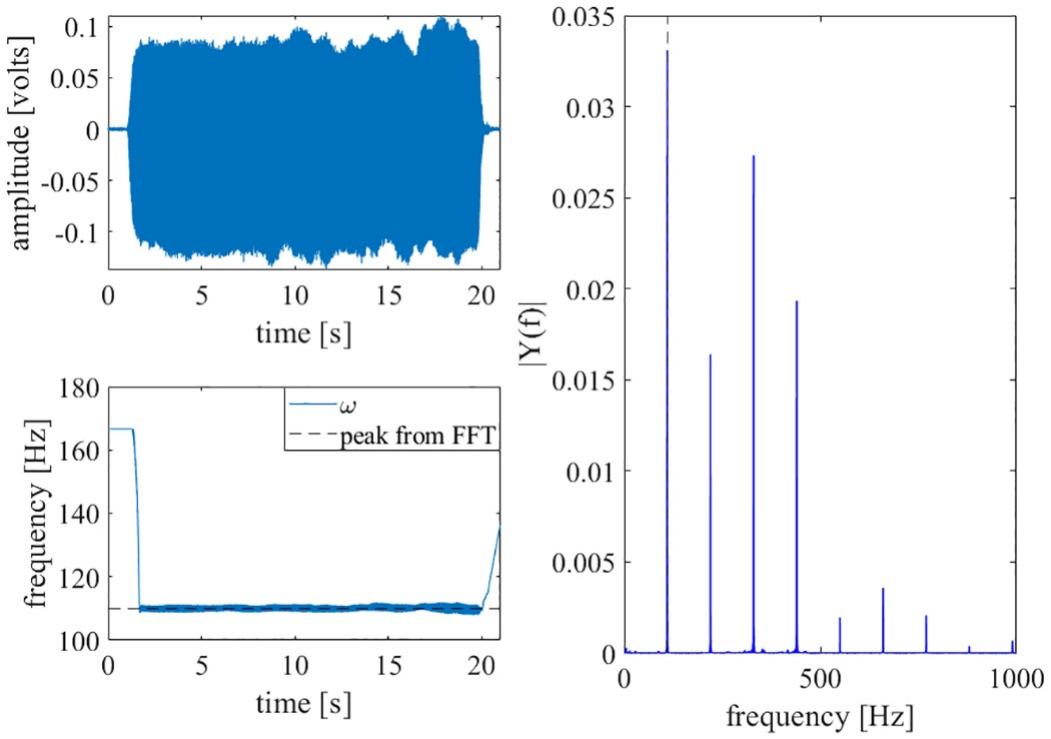
Robotic player trumpet results. A scaled recording of a robotic player trumpet was input to the adaptive oscillator circuit. The circuit learned the natural frequency of the trumpet’s note. Top-left: The time history of the trumpet recording is shown. The time was rescaled by a factor of four. Bottom-left: The circuit learns the first natural frequency of the note. Right: An FFT of the note is shown, with the first natural frequency marked by a dashed, vertical line.

Last, a robotic player trumpet playing an *A* − 440 note (440 Hz) was recorded. This note was then rescaled in time to produce a 110 Hz note. Even though the trumpet’s timbre has higher harmonics, the adaptive oscillator is still able to learn the fundamental frequency. The results are shown in Fig 10.

## 7 Conclusion

In this paper, an adaptive oscillator was designed, fabricated, and tested. SPICE simulations were compared to the experimental results. The results collected from the PCB show qualitatively similar behavior to the adaptive oscillator’s simulated equations. The four-state oscillator was shown to learn the forcing frequency successfully from approximately 25 Hz to 175 Hz within 3% error and input amplitudes from approximately 0 to 3 within 2% error. These experimental results were in close agreement with the SPICE simulation results. There are two types of nonlinearity in this paper. The nonlinearities of the ODEs are desired, and they cause the learning to happen. The circuit nonlinearities (e.g., parasitic elements, etc.) are not modeled, and these nonlinearities cause errors in the experimental results.

As demonstrated in Figs 6–10, this adaptive frequency oscillator is capable of learning more complex waveforms, in addition to simple sinusoids. As demonstrated in Fig 8, this oscillator is capable of reporting the first natural frequency of a mechanical system, without the need of a fast Fourier transform or other post-processing techniques. The implementation presented here could be used as an analog controller for walking robots, a circuit component of clock oscillators, an analog signal analyzer, or a component of a electromechanical energy harvester (such as that presented in [35]).

It should also be mentioned that other types of implementations of adaptive oscillators could be achieved. For example, adaptive oscillators could likely be implemented in a field programmable analog array, such as the implementation of a chaotic oscillator in [36]. The circuit presented in this paper is a prototype system that is a physical embodiment of Eq 3, which could be further refined for specific, practical applications.

It should be noted that this design may be scaled in frequency via the capacitors used in the integrator circuits. This scaling creates tuning possibilities for analog adaptive oscillators’ frequency-learning range from tens of Hz to MHz. Expanding on the demonstrated capabilities, an array of these circuits could perform a wide range of signal processing capabilities for real-life hardware.

## Supporting information

S1 Fig Full schematic of circuit. Full circuit schematic for the four-state adaptive system, with states *V*_*x*_, *V*_*y*_, *V*_*ω*_, and *V*_*α*_. And the DC power supply used here is +15 and −15 volts, in addition, all unmarked resistors are 1kΩ.
(TIF)
