# Supplementary figures and images for: A four-state adaptive Hopf oscillator
Source: PLoS One. 2021 Mar 25;16(3):e0249131. doi: 10.1371/journal.pone.0249131 (PMC7993838; doi:10.1371/journal.pone.0249131)

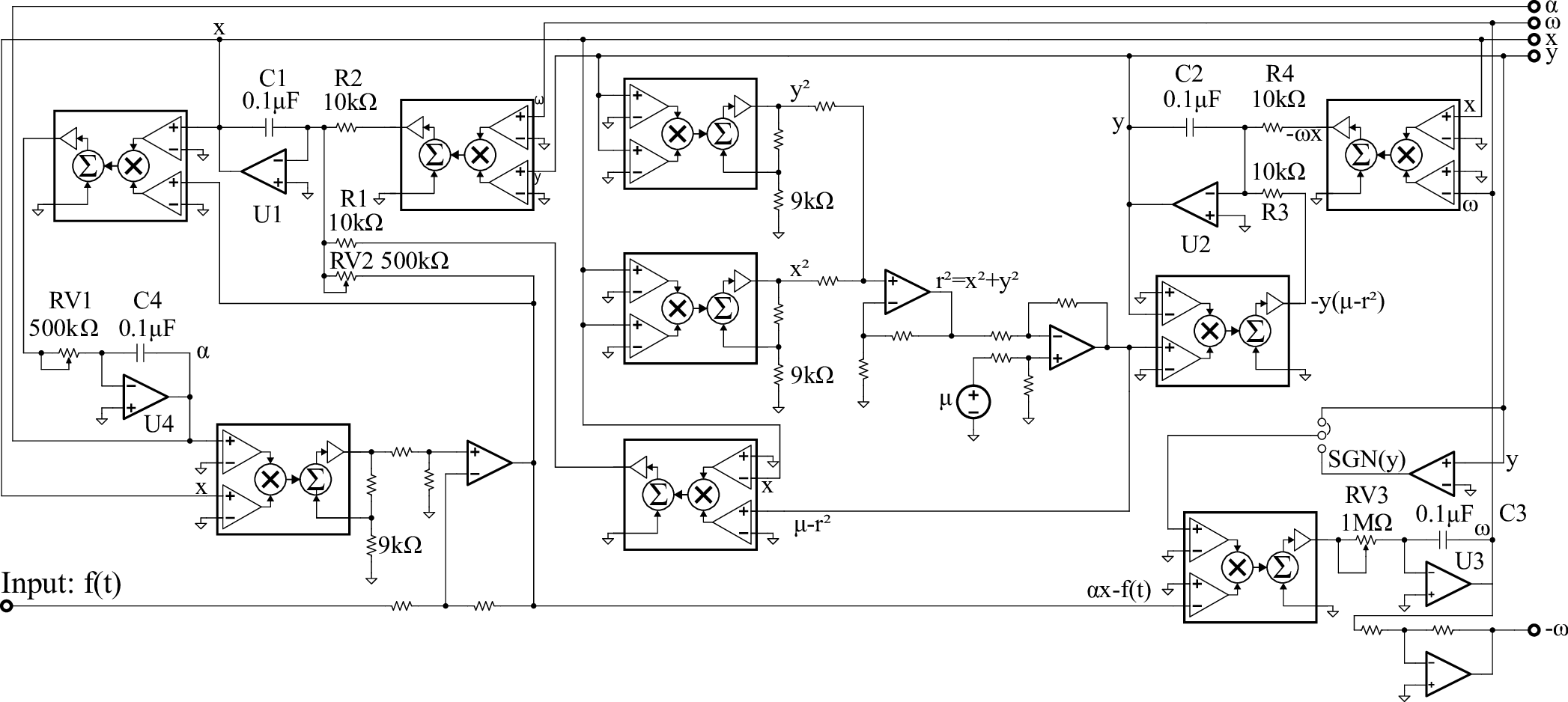

Supplement: S1 Fig — Full circuit schematic for the four-state adaptive system, with states Vx, Vy, Vω, and Vα. And the DC power supply used here is +15 and −15 volts, in addition, all unmarked resistors are 1kΩ. (TIF) [file pone.0249131.s001.tif]
